# The autoinducer synthases LuxI and AinS are responsible for temperature-dependent AHL production in the fish pathogen *Aliivibrio salmonicida*

**DOI:** 10.1186/s12866-015-0402-z

**Published:** 2015-03-24

**Authors:** Hilde Hansen, Amit Anand Purohit, Hanna-Kirsti S Leiros, Jostein A Johansen, Stefanie J Kellermann, Ane Mohn Bjelland, Nils Peder Willassen

**Affiliations:** Norwegian Structural Biology Centre and the Department of Chemistry, Faculty of Science and Technology, UiT The Arctic University of Norway, N-9037 Tromsø, Norway; Section for Microbiology, Immunology and Parasitology, Department of Food Safety and Infection Biology, Faculty of Veterinary Medicine and Biosciences, Norwegian University of Life Sciences, Akershus, Norway; Current address: Department of Chemistry and Pharmacy, Institute of Biochemistry, University of Münster, Wilhelm-Klemm-Straße 2, 48149 Münster, Germany

**Keywords:** *Aliivibrio salmonicida*, Quorum sensing, Biofilm, Acyl homoserine lactone, Temperature

## Abstract

**Background:**

Quorum sensing (QS) is a cell-to-cell communication system used by bacteria to regulate activities such as virulence, bioluminescence and biofilm formation. The most common QS signals in Gram-negative bacteria are N-acyl-homoserine lactones (AHLs). *Aliivibrio salmonicida* is the etiological agent of cold water vibriosis in Atlantic salmon, a disease which occurs mainly during seasons when the seawater is below 12°C. In this work we have constructed several mutants of *A. salmonicida* LFI1238 in order to study the LuxI/LuxR and AinS/AinR QS systems with respect to AHL production and biofilm formation.

**Results:**

Using high-performance liquid chromatography-tandem mass spectrometry (HPLC-MS/MS) we found that LuxI in *A. salmonicida* LFI1238 is responsible for producing seven of the different AHLs, whereas AinS is responsible for producing only one. The production of these various AHLs is dependent on both cell density and growth temperature. The AHLs were efficiently produced when wild type LFI1238 was grown at 6 or 12°C, however at 16°C AHL production decreased dramatically, and LFI1238 produced less than 5% of the maximum concentrations observed at 6°C. LitR, the master regulator of QS, was found to be a positive regulator of AinS-dependent AHL production, and to a lesser extent LuxI-dependent AHL production. This implies a connection between the two systems, and both systems were found to be involved in regulation of biofilm formation. Finally, inactivation of either *luxR1* or *luxR2* in the *lux* operon significantly reduced production of LuxI-produced AHLs.

**Conclusion:**

LuxI and AinS are the autoinducer synthases responsible for the eight AHLs in *A. salmonicida.* AHL production is highly dependent on growth temperature, and a significant decrease was observed when the bacterium was grown at a temperature above its limit for disease outbreak. Numerous AHLs could offer the opportunity for fine-tuning responses to changes in the environment.

**Electronic supplementary material:**

The online version of this article (doi:10.1186/s12866-015-0402-z) contains supplementary material, which is available to authorized users.

## Background

Members of the *Vibrionaceae* family are found in a wide range of aquatic environments, as free-living planktonic cells or attached to surfaces as bacterial aggregates or biofilms. Some members within this family have evolved to become pathogens whereas others have developed symbiotic relationships with their hosts [1]. *Aliivibrio salmonicida*, previously designated *Vibrio salmonicida* [2], is the causative agent of cold water vibriosis in Atlantic salmon (*Salmo salar*), rainbow trout (*Oncorhynchus mykiss*) and captive Atlantic cod (*Gadus morhua*) [3-5], a disease which is kept under control due to successful vaccination [6]. The mechanisms of *A. salmonicida* virulence and pathogenicity are not known in detail, however, temperature-sensitive iron sequestration, motility and flagella activity, as well as quorum sensing (QS) have been suggested to be involved in virulence [7-10]. Moreover, the genome of *A. salmonicida* LFI1238 encodes putative hemolysins, proteases and several protein secretion systems that may be found to be important in pathogenesis [11].

Bacteria use QS to regulate and coordinate gene expression by secreting and responding to signal molecules in a cell density dependent manner [12]. The most common QS autoinducer signal molecules in Gram-negative bacteria are N-acyl homoserines lactones (AHLs), which consist of a homoserine lactone (HSL) linked to an acyl side chain with 4–18 carbons [13-16]. Several QS systems have been identified in vibrios and aliivibrios, and each species seems to utilize a unique combination of QS systems that can act in parallel, or in a hierarchal manner to regulate species-specific activities including biofilm formation, virulence and colonization factors [17,18]. The well-studied squid symbiont *Aliivibrio (Vibrio) fischeri,* a close relative of *A. salmonicida,* regulates bioluminescence and colonization by the QS systems LuxS/LuxPQ, AinS/AinR and LuxI/LuxR, where LuxS, AinS and LuxI are the autoinducer synthases [17,19-21]. AinS in *A. fischeri* produces N-octanoyl-HSL (C8-HSL) which is recognized by the membrane-bound two-component hybrid sensor kinase AinR [22,23]. LuxS in *A. fischeri* does not produce AHLs, but instead makes a signal molecule referred to as autoinducer 2 (AI-2) [20] that likely binds the periplasmic receptor LuxP and modulates the activity of the sensor kinase LuxQ, similar to the homologous system in *Vibrio harveyi* [24]. The LuxS/LuxPQ and AinS/AinR systems in *A. fischeri* work in parallel, and in the absence of signal molecules LuxPQ and AinR are believed to function as kinases and phosphorylate the response regulator LuxO via LuxU [17,18,20]. Phosphorylated LuxO then activates expression of *qrr* which encodes a small regulatory RNA. Qrr destabilizes the *litR* mRNA that encodes the master regulator of QS. In the presence of signal molecules at high cell density, LuxO becomes dephosphorylated leading to suppression of *qrr* allowing LitR to be expressed [17,25,26]. The third system in *A. fischeri,* LuxI/LuxR, is activated by the AinS/AinR system, and at medium cell densities LuxR binds AinS-produced C8-HSL and activates transcription of the *lux* operon (consisting of the *luxICDABEG* and *luxR* loci). At higher cell densities, LuxI-produced N-3-oxo-hexanoyl-L-HSL (3-oxo-C6-HSL) binds to LuxR and increases transcription of the *luxICDABEG* and *luxR* genes [27,28]. Moreover, the master regulator LitR is a positive regulator of *ainS* and is indirectly involved in expression of *luxI* by controlling expression of *luxR.* Hence, LitR links the AinS/AinR and LuxS/LuxPQ systems to the LuxI/LuxR system in *A. fischeri* [20,25].

The number and types of AHLs vary between different species of *Vibrio* and *Aliivibrio*, as well as between strains of the same species [29-34]. However, the specific autoinducer synthases responsible for the different AHLs are only known for a few bacteria, such as the above mentioned in *A. fischeri*. In *V. anguillarum*, another fish pathogen, the AinS homolog VanM is reported to produce N-3-hydroxy-hexanoyl-HSL (3-OH-C6-HSL) and N-hexanoyl-HSL (C6-HSL) [35], whereas the LuxI homolog VanI produces N-3-oxo-decanoyl-HSL (3-oxo-C10-HSL) [36]. In the bioluminescent bacteria *V. harveyi* the LuxM synthase is reported to produce N-3-hydroxy-butyryl-L-HSL (3-OH-C4-HSL) [37,38]. Thus, while some autoinducer synthases produce only one AHL others are able to produce several.

The genome of *A. salmonicida* LFl1238 encodes five QS systems: the AinS/AinR, LuxI/LuxR, VarS/VarA, LuxM/LuxN and LuxS/LuxPQ systems. The latter two systems are probably inactive since *luxM* is absent, and *luxN* and *luxP* contain frame-shift mutations [11]. The *lux* operon of *A. salmonicida* has a novel organization compared to *A. fischeri,* with two flanking *luxR* genes (*luxR1* and *luxR2*) and the *luxI* gene located outside the *luxCDABEG* locus [39], and *A. salmonicida* is only able to produce bioluminescence after addition of decyl aldehyde [40]. Deletion of *luxA* in the *lux* operon of *A. salmonicida* has been associated with decreased virulence [39]. The master regulator of QS, LitR, is also involved in regulation of virulence in *A. salmonicida* LFl1238 as well as biofilm formation, motility and cryptic bioluminescence [10,41].

In recent work we identified and quantified AHLs in 57 members of the *Vibrionaceae* family using high-performance liquid chromatography combined with mass spectrometry (HPLC-MS/MS) [34]. Mapping of the resulting AHL profiles onto a host 16S rDNA phylogenetic tree revealed that closely related strains produce similar AHL profiles. One of the 57 isolates included was *A. salmonicida* strain LFI1238, from which we were able to identify a total of eight different AHLs. The AHLs were analyzed at only one cell density from cultures grown at one temperature in this study [34]. Since in *A. salmonicida* the regulatory role of LitR on some phenotypes is temperature dependent [10,41] we wanted to analyze the impact of temperature on AHL production. Hence, in the work presented here we have analyzed the AHL profiles of *A. salmonicida* wild type LFI1238 and a Δ*litR* mutant at different cell densities during growth at three different temperatures. We have also inactivated the AHL synthetases AinS and LuxI in order to identify which synthetase makes which AHL(s). Finally, we studied AHL production in *luxR* mutants, and analyzed the involvement of the AinS/AinR and LuxI/LuxR systems in biofilm formation.

## Methods

### Chemicals and AHL standards

The following AHL standards were purchased from University of Nottingham, UK: N-3-oxo-butyryl-L-homoserine lactone (3-oxo-C4-HSL), N-3-hydroxy-butyryl-L-homoserine lactone (3-OH-C4-HSL), N-3-hydroxy-hexanoyl-L-homoserine lactone (3-OH-C6-HSL), N-3-hydroxy-octanoyl-L-homoserine lactone (3-OH-C8-HSL), N-3-hydroxy-decanoyl-L-homoserine lactone (3-OH-C10-HSL). Standards purchased from Sigma-Aldrich were: N-butyryl-DL-homoserine lactone (C4-HSL), N-hexanoyl-L-homoserine lactone (C6-HSL), N-3-oxo-hexanoyl-L- homoserine lactone (3-oxo-C6-HSL), N-octanoyl-L-homoserine lactone (C8-HSL), N-3-oxo-octanoyl-L-homoserine lactone (3-oxo-C8-HSL), N-decanoyl-DL-homoserine lactone (C10-HSL), N-3-oxo-decanoyl-L-homoserine lactone (3-oxo-C10-HSL), N-dodecanoyl-DL-homoserine lactone (C12-HSL), N-3-oxo-dodecanoyl-L-homoserine lactone (3-oxo-C12-HSL), and N-3-hydroxy-dodecanoyl-DL-homoserine lactone (3-OH-C12-HSL). HPLC grade acetonitrile and formic acid were purchased from Merck.

### Strains and culture conditions

Bacterial strains and plasmids used in this study are listed in Table 1. The *A. salmonicida* strains were grown at 12°C in either Lysogeny Broth (LB) or Tryptic Soy Broth (TSB) (Difco, BD Diagnostics) supplemented with 2.5% or 1.5% NaCl, respectively. The *Escherichia coli* strains S17.1 and DH5α were cultured in LB medium, whereas DH5αλ*pir* was cultivated in Brain Heart Infusion (BHI) (Oxoid, Cambridge, UK) medium at 37°C. The plasmids pDM4 and pNQ705 were propagated in S17.1 cells [42,43] and pEVS122 and pEVS104 were propagated in DH5αλ*pir* and DH5α, respectively [44,45].

**Table 1:** **Bacterial strains and plasmids used in this study**

**Bacterial strain or plasmid**		**Description** ^**a**^	**Source or reference**
*A. salmonicida*			
	LFI1238	Wild type, isolated from Atlantic cod	[11]
	Δ*litR*	LFI1238 containing an in-frame deletion of *litR*	[10]
	Δ*ainS*	LFI1238 containing an in-frame deletion of *ainS*	This study
	Δ*ainS/luxI ˉ*	Δ*ainS* with insertional disruption of *luxI*; Cm^r^	This study
	*luxI ˉ*	LFI1238 with insertional disruption of *luxI*; Cm^r^	This study
	*luxR1ˉ*	LFI1238 with insertional disruption of *luxR1* gene, Erm^r^	This study
	*luxR2ˉ*	LFI1238 with insertional disruption of *luxR2* gene, Erm^r^	This study
*E. coli*			
	S17-1	Donor strain for conjugation, λ-pir	[42]
	DH5αλ *pir*	Donor strain for conjugation	[45]
	DH5α	Helper strain for conjugation	Invitrogen
*Plasmids*			
	pDM4	Cm^r^; suicide vector with an R6K origin (λ-pir requiring) and *sacBR*	[43]
	pNQ705	Cm^r^; suicide vector with an R6K origin (λ-pir requiring)	[43]
	pEVS122	Erm^r^; suicide vector with an R6Kγ *oriV, oriT*_*RP4*_, *lacZ*α*,cosN, loxP, incD*	[45]
	pEVS104	Helper plasmid	[44]
	pDM4Δ*ainS*	pDM4 containing a fragment of *ainS* harbouring an internal deletion	This study
	pNQ705*luxI*	pNQ705 containing a 245 bp fragment of *luxI*	This study
	pEVS*luxR1*	pEVS122 containing a 224 bp fragment of *luxR1*	This study
	pEVS*luxR2*	pEVS122 containing a 280 bp fragment of *luxR2*	This study

^a^Cm^r^, Chloremphenicol resistance gene. Erm^r^, Erythromycin resistance gene.

*E. coli* carrying pNQ705 and pDM4 constructs were grown on media containing 25 μg/ml chloramphenicol, whereas 100 μg/ml kanamycin or 250 μg/ml erythromycin were used in the media for propagating *E. coli* carrying pEVS104 and pEVS122. *A. salmonicida* transconjugants were selected on LB agar plates containing 2.5% NaCl and 2 μg/ml chloramphenicol, or on TSB agar plates containing 1.5% NaCl and 25 μg/ml erythromycin.

### DNA extraction, PCR and sequencing

Extraction of DNA, recombinant DNA techniques and transformations were performed according to standard protocols [46]. Restriction enzyme digestion, ligation and plasmid purification were performed as recommended by the manufacturers (NEB Biolabs, OMEGA Bio-Tek and Invitrogen). PCR using Phusion (FinnZyme) or Taq polymerase (Biolabs) as well as Big Dye sequencing (Applied Biosystems) were performed with custom made primers (Sigma, Operon and Medprobe). The primers used for PCR and sequencing are listed in Additional file 1: Table S1.

### Construction of *A. salmonicida* LFI1238 mutants

Construction of the Δ*litR* in-frame mutant has been described elsewhere [10]. Similarly, the *ainS* gene was deleted in *A. salmonicida* by allelic exchange. In brief, the sequences flanking *ainS* were amplified by PCR from genomic DNA of LFI1238. The A and B primers were used for amplification of the region upstream *ainS*, and primers C and D for amplification of the downstream region (Additional file 1: Table S1). Primers B and C contain complementary sequences that enable fusion of the upstream and the downstream PCR products by an overlap PCR using the outermost primers A and D. This results in a removal of 357 codons (including the start codon) from the *ainS* open reading frame, and hence, only 40 codons remain. The primers A and D contain *Spe*I and *Xho*I restriction enzyme sites, respectively, in their 5’end. The overlapping PCR product was digested with *Spe*I and *Xho*I, ligated into the corresponding sites of the suicide vector pDM4 and transformed directly into *E. coli* S17-1 cells.

The *luxI* gene was inactivated by plasmid insertion in the wild type *A. salmonicida* LFI1238 as well as in the Δ*ainS* mutant. The plasmid used for insertional inactivation was made by amplifying an internal part of *luxI* (245 bp) by PCR. Adenine overhangs were added to the PCR products using a Taq polymerase before being ligated into the pGEM T-Easy vector (Invitrogen) and transformed into *E. coli* DH5α. The resulting plasmid was purified, digested with *Spe*I and *Xho*I and finally the re-purified PCR products were cloned into the corresponding sites of the suicide vector pNQ705. Similarly, internal fragments of *luxR1* (224 bp) and *luxR2* (280 bp) were PCR amplified and cloned into a pCR™4-TOPO® TA cloning vector according to the manufacturer’s instructions (Invitrogen). The *luxR1* and *luxR2* fragments were cut from the resulting plasmids using *Bam*HI and cloned into the corresponding sites of pEVS122.

The pDM4 and pNQ705 constructs were transformed into *E. coli* S17-1 and used as donors in mating experiments with their respective parental *A. salmonicida* strain as described previously [10]. For inactivation of *luxR1* and *luxR2* tri-parental mating was performed [39,45]. To this end, pEVS*luxR1* or pEVS*luxR2* was transferred from *E. coli* DH5αλ*pir* to *A. salmonicida* LFI1238 using pEVS104 contained in *E. coli* DH5α as helper plasmid. Briefly, DH5αλ*pir*, DH5α and LFI1238 were grown to their stationary phases. In this experiment the LFI1238 was grown at 15°C in TSB containing 1.5% NaCl. The volume of each culture was adjusted to account for a 1:1:1 ratio of cells. The cells were pelleted by centrifugation, washed twice and combined by re-suspension in chilled TSB before being spotted onto chilled blood agar plates with 2.5% NaCl. The plates were incubated at 21°C for 5–6 hours followed by incubation at 15°C for 16 hours. The resulting confluent growth of cells was re-suspended in chilled TSB and cultivated for 24 hours at 12°C with 200 rpm. Finally, the suspension was plated onto TSB agar plates with 1.5% NaCl and erythromycin. After 5–7 days of growth, erythromycin resistant *luxR1* and *luxR2* mutants were isolated.

### Preparation of bacterial supernatants for AHL measurements

Two or more biological replicates were used for all *A. salmonicida* strains, with three technical replicates of each sample collected at different time points. The primary cultures (2 ml) were grown from individual colonies in LB medium with 2.5% NaCl at 12°C with 220 rpm. After 48 hours, a secondary culture was made by diluting the primary culture 1:20. The secondary cultures were grown 24 hours before being diluted to OD_600_ = 0.001 or 0.050 (optical density measured at 600 nm) in a total volume of 60 ml LB with 2.5% NaCl. The cultures were grown further in 250 ml baffled flask at 220 rpm. Samples (1 ml) were harvested at regular intervals and centrifuged at 17000 g for 1 minute (Heraeus Fresco 21, Thermo Scientific). The supernatants were acidified before ethyl acetate extraction as previously described [34]. The ethyl acetate phase was dried using a rotary vacuum centrifuge (PH40-11, Savant Instruments Inc.) and re-dissolved in 150 μl of 20% acetonitrile containing 0.1% formic acid and 775 nM of the internal standard 3-oxo-C12-HSL.

Samples from three separate experiments were harvested and prepared as described above. In the first experiment, the secondary cultures of LFI1238 and Δ*litR* were diluted to an OD_600_ of 0.050 and grown at 6, 12 and 16°C. Here, the samples were harvested at eight different time points starting from an OD_600_ of ~ 0.5 through to the stationary phase. In the second experiment, cultures of LFI1238, Δ*ainS*, *luxIˉ* and Δ*ainS/luxIˉ* were started at an OD_600_ of 0.001, grown at 12°C and harvested after 50 hours. In the third experiment the different cultures (LFI1238, *luxR1ˉ and luxR2ˉ)* were started at an OD_600_ of 0.001 and grown at 12°C before samples were collected at an OD_600_ of 1.8 (approx. 36 hours). The AHL molecules were separated by HPLC before being identified using MS/MS with selective reaction monitoring (SRM) (first and second experiments) or using a Full Scan High Resolution (HR) MS method (see below).

### Detection of AHL profiles using HPLC-MS/MS analysis

The HPLC-MS/MS analysis was performed as previously described [34]. In brief, HPLC was performed using a Hypersil GOLD C18 reverse phase column (50 × 2.1 mm, 1.9 μm particle size, Thermo Scientific) and eluted with a 162 second gradient of 5-95% acetonitrile in 0.1% formic acid at a flow rate of 500 μl/min. The LTQ (Linear Ion Trap Quadrupole) part of the LTQ orbitrap XL (Thermo Scientific) was used in SRM mode for detection of fragment m/z 102 from parent ions. The retention time was divided into 6 segments of varying lengths with two to three scan events for each segment. The data was collected from 101 to 103 m/z.

### Detection of AHL profiles using HPLC with Full Scan HR-MS analysis

HPLC with Full Scan HR-MS was performed using the LTQ Orbitrap XL and Accela Autosampler (Thermo Scientific). The samples (20 μl) were injected onto the same reverse phase column as described above. The elution procedure was performed with an acetonitrile gradient in 0.1% formic acid, and consisted of 5% acetonitrile for 18 seconds, followed by a linear gradient up to 90% acetonitrile over 402 seconds, and finally 90% acetonitrile for 150 seconds. The column was re-equilibrated for 150 seconds with 5% acetonitrile in 0.1% formic acid before the next sample was injected. The flow rate was 200 μl/min for all steps. The separated compounds were detected under positive ion conditions by electrospray ionization using the following settings: sheath gas flow rate 35, auxiliary gas flow rate 20, sweep gas flow rate 0, spray voltage +4.50 kV, capillary temperature 300°C, capillary voltage 47 V, and tube lens 80–90 V. The orbitrap was operated in the full scan mode from m/z 165–450 at a resolution of 15.000 with target setting of 5 × 10^5^ ions per scan and collection of data in the profile mode. The maximum ion injection time was 500 ms. Lock mass was enabled for correction of background ions from caffeine (m/z 195.0877) and diisooctyl phthalate (m/z 391.2843 and m/z 413.2662). The system was calibrated with a mixture of 15 AHLs including the internal standard 3-oxo-C12-HSL, and the ion chromatograms were analyzed using the Xcalibur v. 2.0.7 software package. The mass window was set to 15 parts per million. The limit of detection (LOD) and the limit of quantification (LOQ) for the different AHLs were calculated as previously described [34] and are shown in Additional file 2: Table S2. This method does not allow quantification of C4 chained AHLs.

### Biofilm assay

The biofilm assay was performed mainly as described elsewhere [41]. In brief, the different strains were grown in LB with 2.5% NaCl at 12°C before being diluted to OD_600_ = 0.1 in SWT medium (5 g/l Bacto Peptone (BD), 3 g/l Yeast Extract (Sigma), 28 g/l Marine Sea Salt (Tetra)). A total volume of 300 μl of each dilution was added to wells in a flat bottom non tissue culture treated Falcon 24-well tray (BD Biosciences). The plates were incubated statically at 4°C for 3 days before being stained with 0.1% crystal violet. The air-dried biofilms were dissolved in 96% ethanol (500 μl/well) before the absorbance was read at 590 nm (V_max_ Kinetic Microplate Reader, Molecular Devices). Three biological replicates were used for each strain, and the experiments were repeated several times.

### Statistical analysis

Student’s *t* test was performed to calculate statistical significance (p-values) using the Microsoft Excel 2010 software.

## Results

### AHL signal production in *A. salmonicida* is cell density and temperature dependent

We recently established a method for detection of AHLs using HPLC-MS/MS and reported that *A. salmonicida* LFI1238 produces 8 different AHLs: 3-oxo-C4-HSL, C4-HSL, C6-HSL, 3-oxo-C6-HSL, C8-HSL, 3-oxo-C8-HSL, 3-oxo-C10-HSL and 3-OH-C10-HSL. In this previous study the bacterium was grown at 12°C and the supernatants were harvested and analyzed after the bacterium had reached the stationary phase [34]. Under laboratory conditions, *A. salmonicida* has a growth optimum between 12-16°C in liquid cultures [4]. However, a seawater temperature below 12°C is normally a prerequisite for *A. salmonicida* to cause cold water vibrosis in Atlantic salmon [47], as well as for siderophore production and iron-regulated outer membrane protein expression [7]. Moreover, growth temperatures below 14-16°C are required for the bacteria to express a number of host-bacterium related phenotypes such as adhesion, colony morphology and biofilm formation [10,41]. Therefore, to investigate whether AHL production is also affected by growth temperature, supernatants from the wild type LFI1238 were harvested at different cell densities during growth at 6, 12 and 16°C.

**Figure 1:**
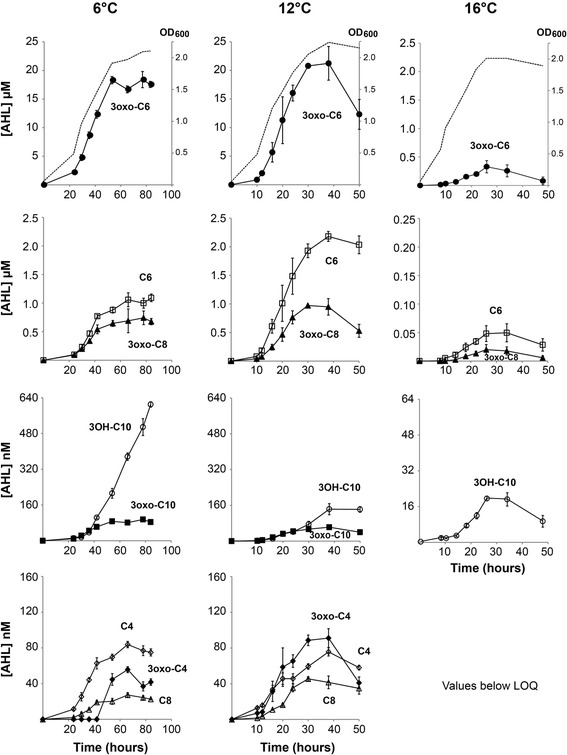
**AHLs produced by** ***A. salmonicida*** **LFI1238 at different temperatures and cell densities.** Supernatants were harvested at different time points during growth at 6, 12 and 16°C. The AHL concentrations were determined by HPLC-MS/MS and are shown with respect to time (hours) and cell density (OD_600_). Symbols indicate the different AHLs: 3-oxo-C6-HSL (●), C6-HSL (□), 3-oxo-C8-HSL (▲), 3-OH-C10-HSL (○), 3-oxo-C10-HSL (■), C4-HSL (◇), 3-oxo-C4-HSL (◆) and C8-HSL (∆). Each value represents the mean of triplicates from three biological replicates. The error bars represent the standard deviations. The dotted lines in the top panels display the growth curves of LFI1238 at different temperatures.

As shown in Figure 1, the different AHLs had reached detectable levels when the measurements started (OD_600_ ~ 0.5), and increased thereafter in a cell density dependent manner. For most AHLs, the highest concentrations were observed near the stationary phase, after which they decreased. Of the dominating AHLs, 3-oxo-C6-HSL reached its maximum concentration (22 μM) after 38 hours growth at 12°C, whereas C6-HSL and 3-oxo-C8-HSL, which were both detected in significantly lower concentrations, reached maxima (2.1 μM and 0.95 μM) after 30 and 38 hours growth respectively, at 12°C. The production of AHLs was highly dependent on temperature, being dramatically lower during growth at 16°C. At this temperature, the maximum concentrations of 3-oxo-C6-HSL, C6-HSL, 3-oxo-C8-HSL and 3-OH-C10-HSL were less than 5% of those produced after growth at 6°C. Only small amounts of 3-oxo-C4-HSL, C4-HSL, C8-HSL and 3-oxo-C10-HSL could be detected at 16°C, and these were at concentrations below the LOQ [34]. Aside from C6-HSL and 3-OH-C10-HSL, the different AHLs were produced at approximately similar levels during growth at 6 and 12°C. The concentration of C6-HSL produced at 12°C was approximately twice that compared to at 6°C. Interestingly, the opposite was observed for 3-OH-C10-HSL where the highest concentration was produced at 6°C, with the concentration rising steadily throughout the experiment reaching a final value of 610 nM compared to only 140 nM after growth at 12°C. Thus, the decrease in growth temperature to 6°C resulted in a four-fold increase of the 3-OH-C10-HSL concentration.

### Identification of AHLs produced by LuxI and AinS

**Figure 2:**
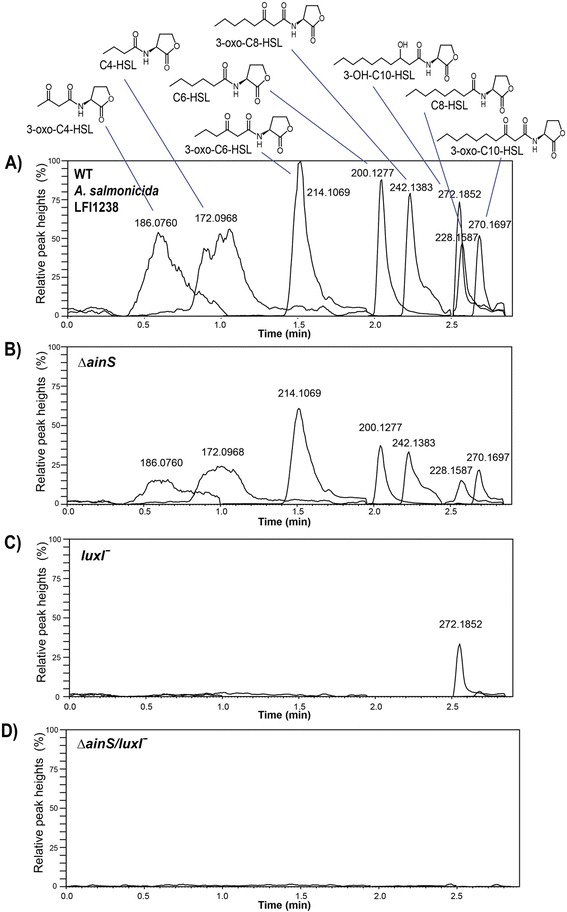
**HPLC-MS/MS ion chromatograms of AHLs produced by** ***A. salmonicida*** **LFI1238 and QS mutants.** **(A)** The wild type LFI1238 produces eight AHLs those being the C4-HSL, 3-oxo-C4-HSL, C6-HSL, 3-oxo-C6-HSL, C8-HSL, 3-oxo-C8-HSL, and 3-oxo-C10-HSL and 3-OH-C10-HSL. **(B)** The Δ*ainS* mutant lacks 3-OH-C10-HSL production, and the chromatogram shows the seven remaining AHLs. **(C)** The *luxIˉ* mutant produces only 3-OH-C10-HSL and **(D)** no AHLs were detected in the supernatants harvested from the double mutant Δ*ainS/ luxIˉ*. The peaks of the different AHLs were scaled so that the different AHLs could be shown in the same chromatogram. The scaling factors were as follows: 1.6 × 10^3^ for 3-oxo-C4-HSL, 6.0 × 10^3^ for C4-HSL, 9.0 × 10^5^ for 3-oxo-C6-HSL, 2.7 × 10^5^ for C6-HSL, 5.4 × 10^4^ for 3-oxo-C8-HSL, 1.5 × 10^4^ for C8-HSL, 1.9 × 10^4^ for 3-OH-C10-HSL and 1.0 x 10^4^ for 3-oxo-C10-HSL. The same scaling was used for all four chromatograms (A, B, C and D). The numbers above the peaks are mass-to-charge ratios (m/z).

Comparison of the genomes of *A. salmonicida* and *A. fischeri*, which are ~65% identical, allowed identification of the genes encoding the AHL synthases LuxI and AinS in *A. salmonicida* LFI1238 [11]. To determine which AHLs are produced by which synthases in LFI1238, the *ainS* and *luxI* genes were interrupted by allelic exchange and plasmid insertion respectively, giving rise to the *∆ainS* and the *luxIˉ* mutants. A double mutant (*∆ainS/luxIˉ*) was also made by interrupting both genes. The wild type LFI1238 and all three mutants were grown at 12°C for 50 hours (OD_600_ ~ 2.2) before samples were collected and analyzed. As shown in Figure 2, LFI1238 produced 8 different AHLs which were identified by their retention times (RT) and mass-to-charge ratio (m/z). The *∆ainS* mutant was unable to produce 3-OH-C10-HSL but the remaining seven AHLs were still detected; whereas the *luxIˉ* mutant produced only 3-OH-C10-HSL, and the double mutant *∆ainS/luxIˉ* showed no AHL production. Thus, the AinS synthase is responsible for the 3-OH-C10-HSL production whereas LuxI is responsible for production of 3-oxo-C4-HSL, C4-HSL, 3-oxo-C6-HSL, C6-HSL, C8-HSL, 3-oxo-C8-HSL and 3-oxo-C10-HSL.

### LitR is a positive regulator of AinS AHL production

**Figure 3:**
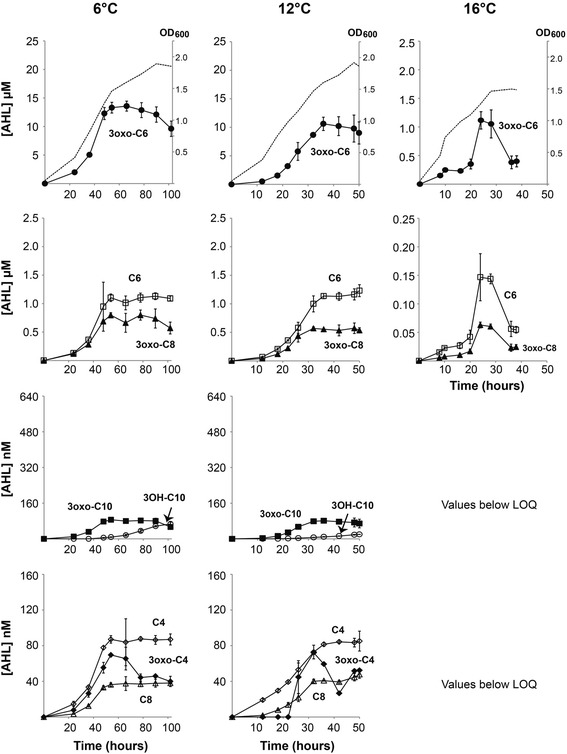
**AHLs produced by the** ***A. salmonicida*** Δ***litR*** **mutant at different temperatures and cell densities.** Supernatants were harvested at different time points during growth at 6, 12 and 16°C. The AHL concentrations were measured by HPLC-MS/MS and are shown with respect to time (hours) and cell density (OD_600_). Symbols indicate the different AHLs; 3-oxo-C6-HSL (●), C6-HSL (□), 3-oxo-C8-HSL (▲), 3-OH-C10-HSL (○), 3-oxo-C10-HSL (■), C4-HSL (◇), 3-oxo-C4-HSL (◆) and C8-HSL (∆). Each value represents the mean of triplicates from three biological replicates. The error bars represent the standard deviations. The dotted lines in the top panels display the growth curves of the Δ*litR* mutant at the different temperatures.

In *A. fischeri* the master regulator LitR is a positive regulator of the LuxI/LuxR and the AinS/AinR systems [20,25]. We therefore wanted to determine if LitR has a similar role in *A. salmonicida*. The *∆litR* mutant was grown at different temperatures as described above and samples were collected throughout the growth curve. In contrast to a previous report [10] the Δ*litR* mutant did not reach to the same cell densities as the wild type LFI1238 in this experiment (Figure 3), which may be due to different culturing conditions or media; however AHL production was still affected by deletion of *litR*. Compared to the wild type LFI1238 (Figure 1) the maximum concentrations of 3-OH-C10-HSL (AinS product) produced by Δ*litR* were only 11% at 6°C and 14% at 12°C (P values < 0.05) (Figure 3), while the amounts of 3-OH-C10-HSL detected after growth at 16°C were below the quantification limit [34]. Similarly, inactivation of *litR* resulted in decreased production of 3-oxo-C6-HSL, C6-HSL and 3-oxo-C8-HSL (LuxI products), and at 12°C the maximum concentrations reached by the *∆litR* mutant were only 50-60% of those reached by LFI1238 (P values < 0.05). The opposite situation was observed when the bacteria were grown at 16°C, where in the absence of *litR* the concentrations of 3-oxo-C6-HSL, C6-HSL and 3-oxo-C8-HSL were approximately two-three times higher than in the wild type. Still, these levels were much lower than those of LFI1238 at 6°C (less than13%). No significant differences in the production of 3-oxo-C4-HSL, C4-HSL, C8-HSL and 3-oxo-C10-HSL were detected between LFI1238 and Δ*litR* at any of the conditions.

### LuxR1 and LuxR2 are required for production of LuxI AHLs

In *A. fischeri*, LuxR and autoinducer activate transcription the *lux* operon encoding the AHL synthetase LuxI and proteins required for bioluminescence [27,28]. We therefore inactivated both copies of *luxR (luxR1 and luxR2)* in *A. salmonicida* LFI1238 by plasmid insertion and analyzed the AHL profiles of the resulting mutants (*luxR1ˉ* and *luxR2ˉ)* using HPLC with Full Scan HR-MS. The wild type and mutants were grown at 12°C and samples collected in the stationary phase. As shown in Table 2, inactivation of either copy caused a significant decrease in the production of LuxI-dependent AHLs, with mutants producing only 5.6% C6-HSL, 3.6% 3-oxo-C6-HSL and 2.7% 3-oxo-C8-HSL relative to wild type LFI1238 concentrations. Small amounts of C8-HSL and 3-oxo-C10-HSL were detected but in concentrations close to or below the quantification limit (Additional file 2: Table S2). Finally, production of the AinS signal (3-OH-C10-HSL) was not affected by mutations in *luxR1* or *luxR2*. It should be noted that the AHL concentrations produced by the wild type were much lower here (Table 2) than detected in the experiment above (see Figure 1). A reason for this could be that the cultures were started at OD_600_ = 0.001 and not at OD_600_ = 0.050 as above. The concentrations of 3-oxo-C4-HSL and C4-HSL were not determined in this experiment.

**Table 2:** **AHL production in** ***A. salmonicida*** **LFI1238 and** ***luxRˉ*** **mutants** ^**a**^

**Bacterial strain**	**C6 (nM)**	**3-oxo-C6 (nM)**	**C8 (nM)**	**3-oxo-C8 (nM)**	**3-oxo-C10 (nM)**	**3-OH-C10 (nM)**
**LFI1238**	359 ± 17	5021 ± 173	15 ± 2	332 ± 27	32 ± 2	53 ± 6
***luxR1ˉ***	20 ± 3	180 ± 51	1.9 ± 0.4	8 ± 1	*	60 ± 1
***luxR2ˉ***	19 ± 5	139 ± 75	*	9 ± 2	*	52 ± 5

^a^Each value represents the mean of triplicates from two biological replicates harvested at OD_600_ ~ 1.8.

The samples were analyzed by the HPLC Full Scan HR-MS method. The concentrations of C4-HSL or 3-oxo-C4-HSL were not determined.

*Below the limit of quantification (LOQ) given in Additional file 2: Table S2.

### The AinS/AinR and LuxI/LuxR pathways regulate biofilm formation

**Figure 4:**
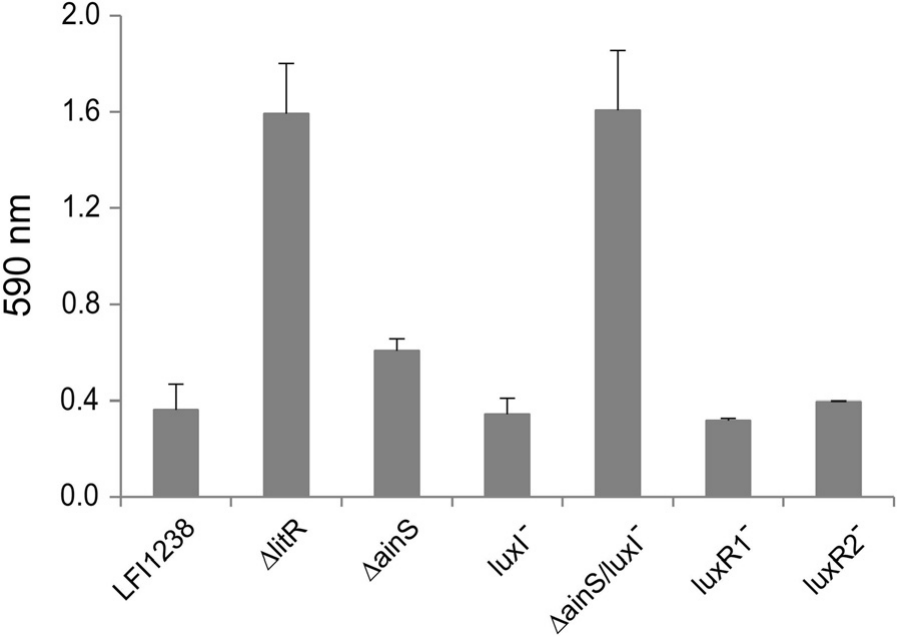
**Biofilm formation of** ***A. salmonicida*** **LFI1238 and QS mutants.** The biofilms were grown statically for 72 hours at 4°C before being stained with crystal violet. The absorbance was read at 590 nm. The error bars represent the standard deviation of three biological replicates.

LitR is a negative regulator of biofilm in *A. salmonicida* LFI1238, hence deletion of *litR* results in increased biofilm formation. The biofilm formation is both temperature- and medium-dependent [10,41]. To establish the impact of AHLs in this process, we analyzed the biofilm formation capabilities of the different mutants. As shown in Figure 4, disruption of either *ainS* or *luxI* alone did not alter biofilm formation, however interruption of both genes simultaneously resulted in a phenotype similar to Δ*litR*. Although *luxR1* and *luxR2* are necessary for manufacture of LuxI-produced AHLs, interruption of these genes did not affect biofilm formation in the assays performed here.

## Discussion

Although *A. salmonicida* is no longer an immediate threat to the aquaculture industry it would be beneficial to understand factors involved in its virulence. Disruption of bacterial communication or QS may present a way to reduce virulence, with the usual strategies being destruction of signals by enzymes, or inactivation by chemicals or natural compounds with antagonistic activities [48].

The relationship between AHL production and cell density is thoroughly documented for a variety of Gram-negative bacteria [18,27]. Similarly, in the study presented here we show that the eight AHLs identified in *A. salmonicida* LFI1238 are also produced in a cell density-dependent manner. *A. salmonicida* was initially reported to produce only two AHLs: C6-HSL and 3-oxo-C6-HSL [30], and reasons for this discrepancy are likely due to different detection methods, strains, growth medium and cultivation temperatures between the different studies. However, by inactivation of two putative AHL synthases in *A. salmonicida* LFI1238 we found that LuxI produces seven AHLs (3-oxo-C4-HSL, C4-HSL, 3-oxo-C6-HSL, C6-HSL, C8-HSL, 3-oxo-C8-HSL and 3-oxo-C10-HSL) whereas AinS only produces one AHL (3-OH-C10-HSL). LuxI homologues in other bacteria are known to catalyze the acylation and lactonization reactions between the substrates S-adenosylmethionine and the acylated ACP (acyl carrier protein) [16,28], thus it is reasonable to assume that LuxI perform the same reaction in *A. salmonicida*. The ability of LuxI to produce an array of different AHLs is intriguing and suggests that this enzyme in *A. salmonicida* LFI1238 does not discriminate well between different substrates, and can accept ACP carrying acyl chains of different lengths as well as acyl chains without or with a keto substitution in the third position (3-oxo). The ability of LuxI homologues to produce a broad spectrum of AHLs is not restricted to *A. salmonicida*. Indeed, the human pathogenic bacteria *Yersinia pseudotuberculosis* has been reported to produce a far greater range of AHLs. This bacterium possesses two LuxI homologous, YpsI amd YtbI, which are responsible for producing over 20 different AHLs containing acyl chains of both odd and even numbers of carbons [49].

The majority of vibrios and aliivibrios produce multiple types of AHLs suggesting that it provides some biological advantages [34]. This was shown for *A. fischeri* where C8-HSL binds and activates both AinR and LuxR at intermediate cell densities, and at higher cell densities LuxR binds 3-oxo-C6-HSL [19]. This sequential activation allows specific regulation of early colonization factors by AinS/AinR, whereas late colonization factors and luminescence are preferentially regulated by LuxI/LuxR. Both systems however are required for persistent colonization of the squid host [21]. Thus, it is possible that *A. salmonicida* exploits its AHL diversity to regulate specific activities in response to temperature or other environmental changes, or during transmission from the seawater into the host and vice versa.

Cold-water vibriosis in Atlantic salmon occurs mainly in late autumn, winter and early spring when the water temperature is below 12°C [47]. We have recently shown that LitR regulates a number of phenotypes, including virulence, in *A. salmonicida* LFI1238 and that this regulation was stronger at lower temperatures [10,41]. Similarly, as shown here, the growth temperature of wild type LFI1238 was found to have a significant impact on AHL production, and increasing the temperature from 12 to 16°C resulted in a drastic decrease of all eight AHLs. This decline cannot be explained by differences in growth rate, since the LFI1238 cultures all reached stationary phase at similar cell densities (OD_600_ ~ 2) irrespective of their growth temperatures. The LuxI AHLs were produced at comparable or higher levels after growth at 12°C compared to 6°C. On the other hand, the AinS signal concentration produced at 6°C was approximately four times higher than at 12°C in LFI1238. This is interesting considering that LitR was found to regulate production of the AinS signal 3-OH-C10-HSL, and the Δ*litR* mutant produced 3-OH-C10-HSL corresponding to ≤ 14% of the wild type concentrations at 6 and 12°C. This finding strengthens the suggestion that LitR exerts its regulatory function(s) more strongly at low temperatures.

**Figure 5:**
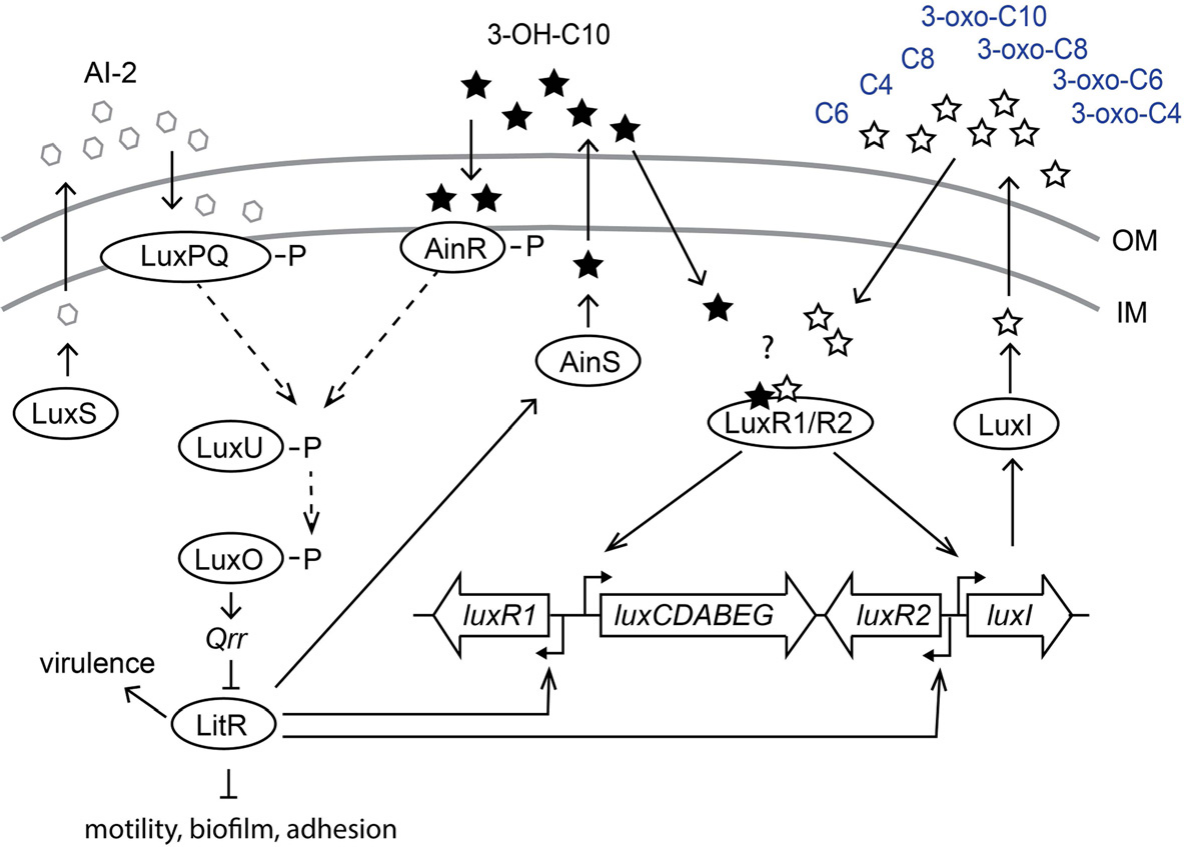
**Illustration of the proposed model of the QS system in** ***A. salmonicida*** **LFI1238.** The autoinducer synthases LuxS, LuxI and AinS, produce the different AHLs and AI-2 which are transported across the inner membrane (IM) and the outer membrane (OM). Their respective receivers are believed to be LuxPQ, a LuxR1-LuxR2 heterodimer, and AinR. The LuxS/LuxPQ pathway may be inactive due to a frame shift mutation within *luxP.* It is unknown which AHLs bind the LuxRs (illustrated with a question mark). At low cell density, a phosphorylation cascade is believed to start from the receivers LuxPQ and AinR, and proceed downstream to LuxO via LuxU (illustrated with dashed arrows). LuxO probably regulates expression of Qrr, which in turn controls the expression of the master regulator LitR. When the autoinducer concentrations are high, LitR is expressed and regulates the production of the AinS AHL, as well as activities such as motility, biofilm, adhesion, virulence and bioluminescence [10]. Both LitR and the LuxRs are probably involved in regulation of the *lux* operon as illustrated.

Similar to LitR in *A. fischeri* [25], our results suggest that LitR also connects the LuxI/LuxR systems to the AinS/AinR and LuxS/LuxPQ systems in *A. salmonicida* (Figure 5)*.* This conclusion is based on the finding that; (i) deletion of *litR* influenced production of LuxI AHLs, (ii) inactivation of *ainS* and *luxI* simultaneously was needed to produce a biofilm similar to the Δ*litR* mutant, as well as our previous results showing that (iii) the *∆litR* mutant produces up to 20-fold less cryptic bioluminescence compared to the wild type LFI1238 [10]. Although LitR is involved in regulation of the *lux* operon, deletion of *litR* does not completely prevent AHL production through LuxI, and the Δ*litR* mutant still produced the LuxI-dependent AHLs 3-oxo-C6-HSL, C6-HSL and 3-oxo-C8-HSL at 50-60% of wild type levels at 6 and 12°C. This suggests that LitR is not essential for *luxR* transcription and that other regulators may be involved in the transcriptional regulation of the two *luxR* genes and hence *luxI.* Autoregulation of *luxR* [50,51], and LitR independent activation of *luxR* have been demonstrated in *A. fischeri*. One of those LitR independent mechanisms involves cAMP and cAMP receptor protein (CRP) [52,53], and more recently the regulatory RNA-binding protein carbon storage regulator A (CsrA) has been shown to increase transcription of *luxR* independently of LitR [54]. *A. salmonicida* LFI1238 encodes the genes for CsrA and CRP [11] and similar mechanisms may therefore explain why AHL production of LuxI is only reduced when *litR* is deleted. On the other hand, our study shows that both LuxRs in *A. salmonicida* LFI1238 are necessary for producing LuxI-AHLs, and inactivation of either *luxR1* or *luxR2* reduced LuxI-produced AHLs significantly without affecting the AinS signal production. A possible explanation for this is that LuxR1 and LuxR2 function as a heterodimer in order to activate transcription of *luxI*, however further studies are needed to elucidate this.

In our work we have studied the role of different QS genes using gene inactivation. It should be pointed out that although our observations with the different mutants are coherent complementation studies for the different mutants would be required to unambiguously prove the function of the inactivated genes.

## Conclusions

In this study we have shown that the AHL autoinducer synthases LuxI and AinS in *A. salmonicida* LFI1238 produced the eight different AHLs. Their production is dependent on both the cell density and cultivation temperature of the bacteria. Production of numerous AHLs suggests that the QS signaling cascade is complex and probably important to fine-tune activities such as virulence, biofilm and other adaption processes to respond to changes in the environment. The AinS/AinR and LuxR/LuxI systems are connected to, and needed for down-regulation of biofilm formation. However, further investigations are needed to understand the regulation and complexity of QS in *A. salmonicida*.

### Ethics statement

The work presented in this paper does not involve human subjects, and we see no ethical issues.

## Additional files

Additional file 1: Table S1. The table lists primers used in this study. Additional file 2: Table S2. The table lists AHL detection limits and quantification parameters for the HPLC Full Scan HR-MS method.

## Abbreviations

AHLs: N-acyl-homoserine lactones
3-OH-C4-HSL: N-3-hydroxy-butyryl HSL
3-OH-C6-HSL: N-3-hydroxy-hexanoyl HSL
3-OH-C8-HSL: N-3-hydroxy-octanoyl HSL
3-OH-C10-HSL: N-3-hydroxy-decanoyl HSL
3-OH-C12-HSL: N-3-hydroxy-dodecanoyl HSL
3-oxo-C4-HSL: N-3-oxo-butyryl HSL
3-oxo-C6-HSL: N-3-oxo-hexanoyl HSL
3-oxo-C8-HSL: N-3-oxo-octanoyl HSL
3-oxo-C10-HSL: N-3-oxo-decanoyl HSL
3-oxo-C12-HSL: N-3-oxo-dodecanoyl HSL
C4-HSL: N-butyryl HSL
C6-HSL: N-hexanoyl HSL
C8-HSL: N-octanoyl HSL
C10-HSL: N-decanoyl HSL
C12-HSL: N-dodecanoyl HSL
HPLC-MS/MS: High-performance liquid chromatography tandem mass spectrometry
HR: High Resolution
m/z: Mass-to-charge ratio
HSL: Homoserine lactone
LB: Lysogeny broth
min: Minutes
OD_600_: Optical density measured at 600 nm
PCR: Polymerase chain reaction
QS: Quorum sensing
rpm: Rounds per minute
SRM: Selective reaction
TSB: Tryptic Soy Broth
