# Supplementary material for: The autoinducer synthases LuxI and AinS are responsible for temperature-dependent AHL production in the fish pathogen Aliivibrio salmonicida
Source: BMC Microbiol. 2015 Mar 24;15:69. doi: 10.1186/s12866-015-0402-z (PMC4377199; doi:10.1186/s12866-015-0402-z)
Supplement: Additional file 1: Table S1. — The table lists primers used in this study. [file 12866_2015_402_MOESM1_ESM.pdf]

Table S1 Primers used in this study [a]

Primer name	Primer sequences (5' - 3')	Source or reference
Primers used for construction of overlap PCR products		
AinS-A	AAT**CTCGAG**AATCACTCTATCCCTCAATCAC	This study
AinS-B	TTTATAAGTGACTAATTTCAGTTAGAGAATCGAAAGTT	This study
AinS-C	TTAGTCACTTATAAAGGTATTTGCT	This study
AinS-D	GGT**ACTAGT**ATTTTAGGATCAACTTTCATC	This study
Primers used for amplification of internal gene fragments		
LuxI-F	**CTCGAG**ATCCGAGTTTACTACTATTCCT	This study
LuxI-R	**ACTAGT**ATCACCAAGTAATTCAGGGA	This study
LuxR1-F	CCC**GGATCC**ACTCACCTATTCTTTGGGACA	This study
LuxR1-R	CCC**GGATCC**GGAAAAGAGACAGTCAGTAGAT	This study
LuxR2-F	CCC**GGATCC**CATCACTCCCCTATTAATTG	This study
LuxR2-R	CCC**GGATCC**TGTGTACCTGTTGATCTTCT	This study
Primers used for verification of plasmids and A. salmonicida mutants		
AinS-E	CCAGCTCGTGCTAATACTTT	This study
AinS-F	GGCAACTCGTTCACTAAAAT	This study
LuxR1-F2	AACCGTCAGATAAAGAGAAACG	This study
LuxR2-F2	AACACCGCTGTACCGTCAAG	This study
LuxI-F2	AAAAAGCAGGCTTCGAAGGAGATAGAACCATGACAATAATGATAAGAAAATCCGAG	This study
LuxI-R2	AGAAAGCTGGGTCTTATATTAATACAGCCTTTTTAAATTG	This study
PNQ-F	TAACGGCAAAAGCACCGCCGGACATCA	Milton et al., 1996
PNQ-R	TGTACACCTTAACACTCGCCTATTGTT	Milton et al., 1996
M13-F	GCCAGGGTTTTCCCAGTCACGA	Invitrogen
M13-R	GAGCGGATAACAATTTCACACAGG	Invitrogen

[a] Nucleotides in bold font are *SpeI* (a/ctagt), *XhoI* (c/tcgag) or *BamHI* (g/gatcc) restriction sites added to the 5' region of the primer
