# Supplementary material for: The autoinducer synthases LuxI and AinS are responsible for temperature-dependent AHL production in the fish pathogen Aliivibrio salmonicida
Source: BMC Microbiol. 2015 Mar 24;15:69. doi: 10.1186/s12866-015-0402-z (PMC4377199; doi:10.1186/s12866-015-0402-z)
Supplement: Additional file 2: Table S2. — The table lists AHL detection limits and quantification parameters for the HPLC Full Scan HR-MS method. [file 12866_2015_402_MOESM2_ESM.pdf]

Table S2 AHL detection and quantification parameters for the HPLC Full Scan HR-MS method

**AHL**[a]	**Parent ion**[b] **$[M + H]^+$ (m/z)**	**Molecular Formula**	**RT**[c] **(min)**	**LOD**[d] **(nM)**	**LOQ**[e] **(nM)**
**C6-HSL**	216.12303	$C_{10}H_{17}NO_4$	4.43	0.92	3.06
**3-oxo-C6-HSL**	214.10738	$C_{10}H_{15}NO_4$	3.02	9.85	32.80
**3-OH-C6-HSL**	200.12812	$C_{10}H_{17}NO_3$	2.94	5.70	18.99
**C8-HSL**	242.13868	$C_{12}H_{19}NO_4$	5.77	0.54	1.81
**3-oxo-C8-HSL**	244.15433	$C_{12}H_{21}NO_4$	4.91	0.89	2.96
**3-OH-C8-HSL**	228.15942	$C_{12}H_{21}NO_3$	4.68	1.59	5.30
**C10-HSL**	270.16998	$C_{14}H_{23}NO_4$	6.86	0.48	1.58
**3-oxo-C10-HSL**	272.18563	$C_{14}H_{25}NO_4$	6.10	0.76	2.54
**3-OH-C10-HSL**	256.19072	$C_{14}H_{25}NO_3$	5.82	0.47	1.57
**C12-HSL**	300.21693	$C_{16}H_{29}NO_4$	7.74	0.45	1.51
**3-oxo-C12-HSL**	298.20128	$C_{16}H_{27}NO_4$	6.98	0.39	1.30
**3-OH-C12-HSL**	284.22202	$C_{16}H_{29}NO_3$	6.72	0.40	1.35

[a] AHL, Acyl homoserine lactones
[b] Monoisotopic mass values calculated using the Xcalibur software version 2.07 (Thermo Scientific)
[c] RT, Retention time, units in minutes
[d] LOD, Limit of detection is defined as the lowest concentration (nM) that can be detected with signal to noise ratio above 3:1
[e] LOQ, Limit of quantification is the concentration equal a signal to noise ratio of 10:1
